# Unbalanced Diets: High-Fat, High-Sucrose and High-Protein Diets

**DOI:** 10.3390/nu17040655

**Published:** 2025-02-12

**Authors:** Bàrbara Reynés, Mariona Palou

**Affiliations:** 1 Laboratory of Molecular Biology, Nutrition and Biotechnology (Nutrigenomics, Biomarkers and Risk Evaluation), University of the Balearic Islands, 07122 Palma, Spain; 2 Health Research Institute of the Balearic Islands (IdISBa), 07010 Palma, Spain; 3 CIBER de Fisiopatología de la Obesidad y Nutrición (CIBEROBN), 28029 Madrid, Spain

This Special Issue of *Nutrients*, “Unbalanced Diets: High-Fat, High-Sucrose and High-Protein Diets” includes five original articles conducted in animal models. These articles address the effects of a cafeteria diet on gut microbiota composition and circulating lipid profiles, the impact of altering the maternal nutritional environment before gestation or during gestation and lactation on the later metabolic health of offspring, and whether interventions during the lactation period can counteract the detrimental effects of unbalanced diets, even in the absence of an excessive caloric intake.

Maintaining a balanced diet is a keystone in achieving and preserving optimal metabolic health [1]. A diet that includes the appropriate proportions of macronutrients carbohydrates, proteins, and fats as well as essential micronutrients, ensures the maintenance of energy homeostasis and overall health [1]. However, modern lifestyles have profoundly disrupted eating habits, making it increasingly challenging to maintain a healthy and balanced diet [2]. In fact, the prevalence of unbalanced diets has increased significantly in recent decades, characterized by an excessive or insufficient intake of specific nutrients, and has been strongly linked to the development of obesity and a myriad of metabolic alterations. These include insulin resistance, dyslipidemia, hypertension, and systemic inflammation, which collectively predispose individuals to chronic diseases such as obesity, type 2 diabetes, cardiovascular disease, cognitive dysfunction, and certain cancers [3].

High-fat (HF) diets have been strongly associated with excessive fat accumulation and homeostasis dysregulation in animal and human studies, leading to different disorders [4,5]. They affect gene expression across key metabolic tissues, upregulating genes associated with catabolic pathways while suppressing those involved in lipogenesis, primarily through insulin, leptin, and nutrients such as glucose and fatty acids signaling pathways [6]. On the other hand, excessive sugar consumption has been linked to obesity, coronary heart disease, type 2 diabetes, metabolic syndrome, and non-alcoholic fatty liver disease, as well as stimulating reward pathways in the brain that may lead to overeating [7]. Thirdly, the Western diet (WD), so-called because of its similarity to the increasingly widespread dietary pattern in the Western world, is a diet high in fat, sugar, and calories. The consumption of a WD has been associated with the incidence of metabolic syndrome [7]. The cafeteria diet (CAFD) is one type of WD, as a high-carbohydrate and HF diet prepared manually, including ultra-processed foods usual in the human diet to mimic the style of the WD [8]. It is a highly palatable diet that induces voluntary hyperphagia, leading to obesity, and is commonly used in studies in rats with diet-induced overweight [8]. In fact, three articles in this Special Issue include this obesogenic diet in their experimental design.

Interestingly, the first article in this Special Issue explores the effects of consuming a CAFD on body weight, microbiota, and lipidemic profiles in adult Wistar rats [9]. Gaza and collaborators showed that a CAFD, which included bacon, biscuits, fried potatoes, a pork pâté base, and liquid chocolate, was rich in palmitic acid, stearic acid, oleic acid, and linoleic acid, with an n-6/n-3 ratio of 34:1 [9]. As expected, CAFD feeding for 15 weeks resulted in obesity and hyperphagia in rats [9]. The CAFD significantly altered the gut microbiota composition of rats, increasing the Firmicutes/Bacteroidetes ratio [9]. The increased ratio of Firmicutes/Bacteroidetes has been considered a biomarker of obesity and gut dysbiosis in some studies, while others have not identified a significant correlation or even a reduced ratio in animals and humans with obesity [10]. Moreover, the results of this paper, showing a lower alpha diversity in the CAFD group compared with controls [9], agrees with the previous research background in humans [11] and rodents [12]. These changes in the composition and diversity are associated with differences in circulating metabolites, which could be further studied as potential biomarkers of this nutritional challenge [9].

Maternal nutrition during critical periods of development such as gestation and lactation plays a pivotal role in shaping the future health of the offspring through a phenomenon known as developmental programming [13]. Maternal obesity is related to a greater predisposition in offspring to develop overweight, and related metabolic alterations such as systemic inflammation and a deterioration in lipid metabolism, in adulthood [14,15]. In this sense, Pomar and collaborators, in the second article in this Special Issue, investigated the effects of maternal CAFD feeding in rats during the lactation period on the metabolic health of their adult offspring, particularly on thermogenesis [16]. The CAFD used, although similar to the version above described by Gaza and collaborators [9], included slight differences, such us including biscuits with a Majorcan sausage (‘sobrassada’), salted peanuts, chocolate, candies, carrots, cheese, sugared milk (20% *w*/*v*) and a Majorcan pastry (‘ensaimada’) [17]. Interestingly, the offspring of CAFD-fed dams exposed to a commercial WD for the last 2 months of the study showed lower expression levels of genes related to lipogenesis, fatty acid uptake, fatty acid oxidation, lipolysis, and thermogenesis, but this was not seen in those fed a standard diet from weaning to the end of the study (6 months old) [16]. These results suggest that a maternal obesogenic diet during the critical developmental period of lactation can alter the programming of the later thermogenic response to a new obesogenic insult in adulthood.

Interventions to counteract or mitigate the malprogramming associated with adverse maternal conditions during gestation and/or lactation represent an emerging area of study, given the high prevalence of women of reproductive age who are following unbalanced diets or who have obesity [18]. In this regard, the next two articles in this Special Issue explore how supplementation with bioactive compounds during lactation in rat dams (cocoa) [19] or suckling pups (leptin and celastrol) [20] could represent strategies to reverse the detrimental programming effects associated with maternal obesity.

Cocoa, the main component of chocolate, is widely consumed and is linked to health benefits, particularly in the prevention of cardiovascular diseases, obesity, and insulin resistance, due to its flavan-3-ols content [21]. However, limited research exists on cocoa’s effects when consumed during pregnancy and lactation on the health of both the mother and offspring. In the third article in this Special Issue, dams supplemented with a cocoa extract rich in flavan-3-ols during lactation showed improved levels of circulating adiponectin and a reduced lipid content in the mammary gland, in accordance with the proposed benefits of cocoa flavanols [19]. Remarkably, the effects on the mothers were transmitted to the offspring, both those fed (from weaning to adulthood) with a standard diet and those under the obesogenic diet, the CAFD already described in [16]. Specifically, these animals also showed a significant increase in adiponectin levels and, moreover, exhibited a reduction in lean/fat ratio and liver weight. In addition, the offspring of dams supplemented with cocoa that followed the CAFD from weaning onwards showed an improvement in the inflammatory profile of their white adipose tissue (WAT) [19]. These results reveal the benefits of moderate cocoa consumption during lactation for both mothers and offspring.

Leptin is a key hormone produced primarily by adipose tissue, modulating satiety and energy expenditure [22], which is also naturally present in maternal milk. It can be absorbed by the immature stomach of rat pups during suckling [23] and program the newborn’s metabolism, influencing the prevention of obesity and metabolic alterations in later stages of life [24]. Celastrol, a plant-derived compound, enhances leptin sensitivity, regulating appetite and energy expenditure [25]. The fourth paper in this Special Issue analyzed in rats the impact of following an HF isocaloric diet from weaning to adulthood and whether leptin and/or celastrol supplementation during the suckling stage can counteract the adverse effects of the unbalanced diet [20]. Rats fed an isocaloric pair-fed HF diet for 11 weeks developed increased adiposity and related metabolic alterations, even without increased body weight. This finding supports that the isocaloric HF feeding in rats results in a metabolically obese, normal-weight (MOWN) phenotype, as previously described [26]. Regarding perinatal treatments, leptin and celastrol resulted in distinct effects in adulthood when rats were exposed to an isocaloric HF diet. Specifically, leptin treatment during lactation prevented liver fat deposition and insulin resistance related to the MOWN phenotype. In the hypothalamus, leptin treatment enhanced insulin and leptin signaling while reducing the expression of certain orexigenic genes. Furthermore, leptin treatment during lactation promoted browning in WAT in MOWN animals. In contrast, celastrol did not exhibit this protective effect and even induced additional metabolic alterations, which were prevented when leptin was co-administered with celastrol. All in all, this study revealed that the perinatal administration of leptin during lactation, but not celastrol, protected against fat accumulation and adiposity-related damage associated with the MOWN phenotype [20].

Finally, maternal diets, even those followed before pregnancy, have been described as having a strong impact on later offspring health [27]. In this sense, the final article in this Special Issue [28] shows how maternal undernutrition before pregnancy results in the malprogramming of later offspring health when exposed to an obesogenic insult. Concretely, protein restriction from puberty (5 weeks old) to adulthood (16 weeks old) in female rats increased the susceptibility of their future offspring to developing metabolic alterations later in life when exposed to an HF diet, particularly insulin resistance, hepatic steatosis and hypercholesterolemia, probably mediated by the hepatic transcriptomic alteration of lipid and glucose metabolism-related genes [28]. These results further expand the long-proposed hypothesis of fetal origins of adult diseases by revealing that maternal—and also paternal—nutrition at any time before or during the critical stages of development can influence metabolic programming in offspring.

To summarize, this Special Issue highlights the profound impact of unbalanced diets, particularly high-fat, high-sugar, and Western style diets, as well as low-protein diets, on metabolic health. Through five studies in animal models, this Special Issue underscores how these diets alter gut microbiota, lipid profiles, and metabolic pathways, contributing to obesity and related disorders. The findings emphasize the critical role of maternal nutrition before gestation and during lactation in programming offspring health, while also exploring potential interventions, such as bioactive compound supplementation, to mitigate adverse effects. One aspect that is not discussed in this Special Issue is the effect of high-protein and ketogenic diets, which are trendy strategies popularly used to control body weight. However, the long-term consequences of following these diets, especially during critical periods of development, remain poorly understood. This issue deserves further discussion. Collectively, this monographic reinforces the importance of balanced diets and targeted strategies to address the growing public health concerns associated with modern dietary patterns.

## Abbreviations

The following abbreviations are used in this manuscript:

CAFD	cafeteria diet
HF	high-fat
MOWN	metabolically obese, normal-weight
WAT	white adipose tissue
WD	Western diet
